# Celiac Crisis: An Unusual Case of Severe Diarrhea

**DOI:** 10.7759/cureus.117701

**Published:** 2026-10-09

**Authors:** Ellie Chen, Vivy Cusumano

**Affiliations:** 1 Gastroenterology, University of California, Los Angeles (UCLA) David Geffen School of Medicine, Los Angeles, USA

**Keywords:** adult celiac crisis, celiac disease, celiac disease (cd), olmesartan-induced enteropathy, refractory diarrhea

## Abstract

Celiac disease is an immune-mediated enteropathy triggered by dietary gluten. Celiac crisis is a severe manifestation characterized by profuse diarrhea, dehydration, hypotension, and severe metabolic derangements. We describe the case of an elderly patient with a remote childhood diagnosis of celiac disease who presented in celiac crisis. A 79-year-old man with hypertension managed on olmesartan presented with two weeks of severe watery diarrhea, weight loss, and edema that progressed to hypotension, acute kidney injury, and profound electrolyte abnormalities, necessitating multiple hospitalizations. Celiac serologies were markedly elevated, and he subsequently disclosed a childhood diagnosis of celiac disease that he had believed resolved. Duodenal biopsies demonstrated duodenitis with intraepithelial lymphocytosis and villous blunting. His symptoms persisted despite a gluten-free diet, withholding olmesartan, and supportive care but improved rapidly after initiation of budesonide. He was diagnosed with celiac crisis, with a possible contribution from olmesartan-associated enteropathy. Repeat endoscopy eight months later showed histologic remission. Celiac crisis should be considered in any patient presenting with severe diarrhea, dehydration, and metabolic abnormalities, especially when there is evidence of underlying celiac disease. In this patient, concurrent olmesartan use raised the possibility of a superimposed drug-induced enteropathy; although olmesartan was discontinued five days before budesonide was started, its contribution cannot be excluded and remains a confounding factor. Even so, this case highlights the importance of promptly recognizing celiac crisis and suggests a potential role for budesonide in patients whose symptoms persist despite gluten withdrawal and supportive care.

## Introduction

Celiac disease is an immune-mediated, multisystem disorder triggered by dietary gluten in genetically susceptible individuals [1]. While most patients are diagnosed with mild-to-moderate gastrointestinal or extraintestinal symptoms, fewer than 1% of patients present with celiac crisis, a fulminant and under-recognized form of the disease marked by severe diarrhea, dehydration, and electrolyte abnormalities [2]. Although more common in children, celiac crisis is increasingly reported in adults, in whom it frequently represents the initial manifestation of previously undiagnosed celiac disease [3]. Olmesartan-associated enteropathy is a sprue-like condition that can produce a similar clinical presentation and histologic findings, complicating the diagnosis [4]. We report the case of a 79-year-old man presenting with celiac crisis, with a possible contribution of olmesartan-associated enteropathy. This case is notable for the patient's advanced age and highlights the importance of recognizing celiac crisis in older adults, particularly given its potential for rapid clinical deterioration and the need for prompt diagnosis and treatment.

## Case presentation

History of present illness

A 79-year-old man with a history of hypertension presented to the clinic for evaluation of acute diarrhea. His symptoms began approximately 1.5-2 weeks prior to presentation, shortly after eating takeout chicken wings. Stools were watery, non-bloody, and occurred up to 10 times daily at onset. He also reported bloating, fecal incontinence, nocturnal bowel movements, lower extremity swelling, and an unintentional 15- to 20-pound weight loss over two weeks. Prior to symptom onset, his bowel habits had been normal. He denied any new medications, recent antibiotic use, hospitalization, sick contacts, travel, or use of nonsteroidal anti-inflammatory drugs (NSAIDs) or proton pump inhibitors.

Past medical, surgical, family, and social history

His past medical history was notable for hypertension and atrial fibrillation status post ablation on apixaban. He had no pertinent surgical history. There was no family history of colon cancer, gastric cancer, inflammatory bowel disease, or celiac disease. Socially, he drank approximately half a bottle of wine daily and denied tobacco use. His outpatient medications were amlodipine 5 mg daily, apixaban 5 mg twice daily, and olmesartan 40 mg daily.

Physical examination

Vital signs were notable for blood pressure 114/66 mmHg, pulse 96 beats per minute, oxygen saturation 97% on room air, and body mass index (BMI) 24.55 kg/m². He was in no apparent distress and appeared his stated age. Cardiopulmonary and neurologic examinations were unremarkable. Abdominal exam revealed a soft, non-tender abdomen with active bowel sounds and a reducible umbilical hernia, without rebound or guarding. Extremities showed trace pitting edema, and skin exam showed no rash or jaundice.

Initial outpatient workup

Initial laboratory studies showed a normal complete blood count and comprehensive metabolic panel and a normal thyroid-stimulating hormone (TSH). Stool infectious testing was all negative. Fecal calprotectin was mildly elevated, and pancreatic elastase was within normal limits (Table 1).

**Table 1:** Laboratory and serological values ^a^ The enteric pathogen panel includes the following microorganisms: *Shigella*, Shiga toxin, *Campylobacter *species, *Salmonella* species. ^b^ The parasite pathogen panel includes *Giardia lamblia*, *Cryptosporidium* species, and *Entamoeba histolytica* *PCR: p*olymerase chain reaction

Laboratory parameter	Outpatient	Initial hospitalization	Second hospitalization	Reference range	Interpretation
Sodium	140	145	144	135-145 mmol/L	-
Potassium	4.1	3.0	4.0	3.6 - 5.3 mmol/L	-
Bicarbonate	20	10	21	20 - 30 mmol/L	-
Magnesium	-	1.5	1.4	1.4 - 1.9 mEq/L	-
Creatinine	1.04	2.14	0.99	0.60 - 1.30 mg/dL	-
Calcium	9.2	8.0	7.6	8.6 - 10.4 mg/dL	-
Albumin	4.0	3.9	3.1	3.9 - 5.0 g/dL	-
Bacterial enteric pathogen panel^a^	Negative	Negative	Negative	Negative	-
*Clostridium difficile* PCR	Negative	-	Negative	Negative	-
Parasite enteric pathogen panel^b^	-	Negative	Negative	Negative	-
Norovirus	-	Negative	Negative	Negative	-
Adenovirus	-	Negative	Negative	Negative	-
Enterovirus	-	Negative	Negative	Negative	-
Fecal calprotectin	150	471	-	<50 µg/g	-
Pancreatic elastase	357	-	-	200 ug/g or greater	-
Tissue transglutaminase IgA	-	1072.4	-	<20.0 CU	Positive
Gliadin IgA	-	769.5	-	<20.0 CU	Positive
Gliadin IgG	-	47.5	-	<20.0 CU	Positive
Endomysial IgA	-	1:80	-	<1:10 titer	Positive

Hospital course - first admission

Six days after his outpatient visit, the patient presented to the emergency department with hypotension. He was afebrile with heart rate 58 beats per minute, blood pressure 94/42 mmHg, and oxygen saturation 99% on room air. Labs now showed significant electrolyte disturbance, metabolic acidosis, and new acute kidney injury (AKI). Additional stool viral infectious studies were negative. Celiac serologies, however, returned strongly positive, including markedly elevated tissue transglutaminase (tTG) immunoglobulin A (IgA), gliadin IgA, gliadin immunoglobulin G (IgG), and endomysial IgA (Table 1). On further questioning, the patient disclosed a childhood diagnosis of celiac disease at eight years of age, which he reported having grown out of, and he had been consuming gluten without apparent difficulty since age 10. He underwent inpatient esophagogastroduodenoscopy (EGD) and colonoscopy.

Endoscopic and Histologic Findings

EGD showed no esophagitis, a 2 cm sliding hiatal hernia with a Schatzki's ring, mild body gastropathy with punctate erythema, and a normal duodenal bulb with possible mild villous blunting in the second portion (Figure 1). Colonoscopy was limited by poor bowel preparation with adherent stool but showed mild loss of vascularity and patchy surface erythema throughout the colon without ulceration, erosion, or friability; the terminal ileum was normal (Figure 2). Biopsies were obtained throughout.

**Figure 1:**
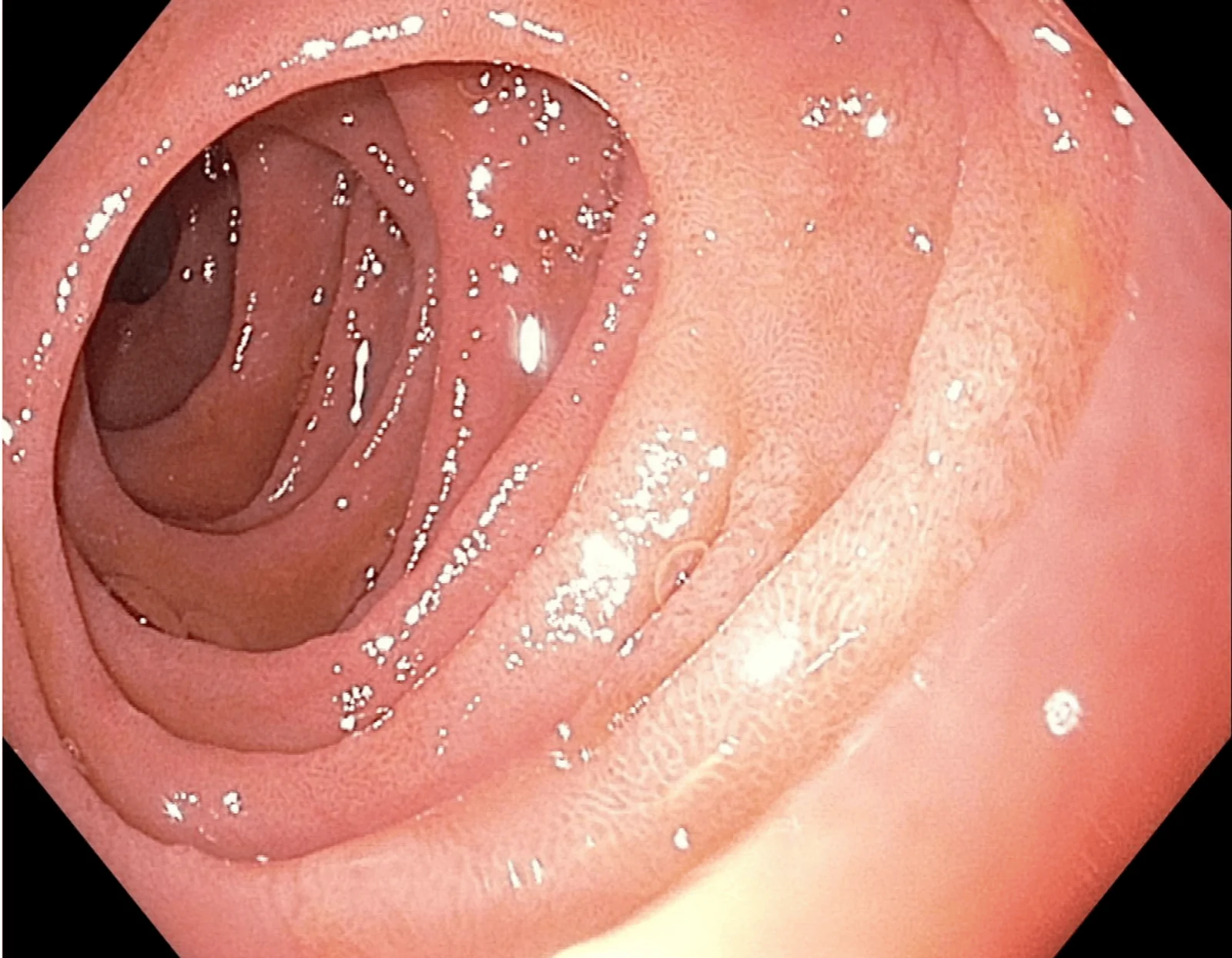
EGD view of the second portion of the duodenum; the mucosal appearance suggests possible villous blunting EGD: esophagogastroduodenoscopy

**Figure 2:**
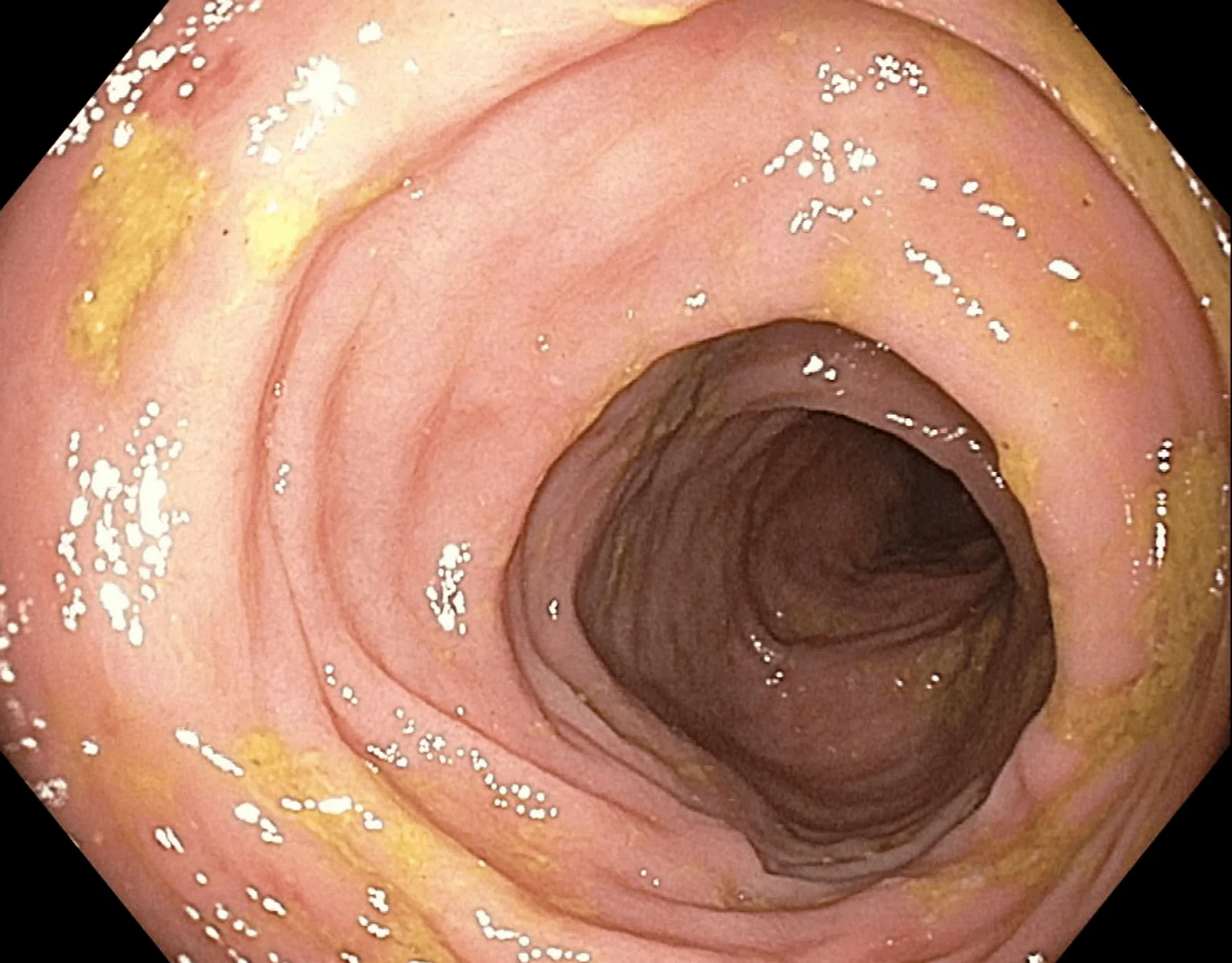
Colonoscopic view of the colon showing mild loss of vascularity and patchy erythema, suggestive of mild colitis.

Pathology showed focal acute duodenitis with intraepithelial lymphocytosis in the duodenum and duodenal bulb, with focal villous blunting in the bulb specimen; mild chronic gastritis without *Helicobacter pylori*; normal terminal ileum; and mildly active colitis on random colonic biopsies. The pathologist noted that the duodenal findings were not specific and could reflect peptic injury, drug-induced injury (including NSAIDs), *H. pylori *gastritis, or infection, while the colonic findings were similarly nonspecific, with infection, medication effect, and ischemia among the considerations (Figure 3).

**Figure 3:**
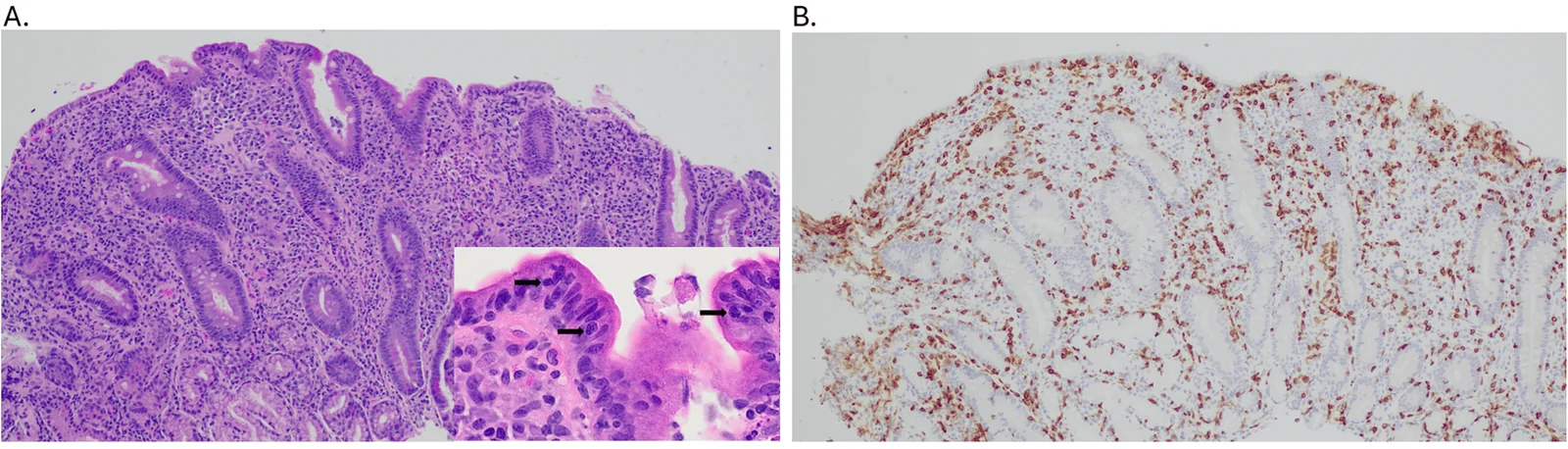
Histology of duodenal biopsies A. Duodenal mucosa with intraepithelial lymphocytosis (arrow) and villous blunting (Hematoxylin and Eosin stain, 100x magnification). B. Immunostain of CD3 (cluster of differentiation 3) highlights T lymphocytes (100× magnification).

Treatment and Discharge

The patient was started on a gluten-free diet along with potassium, bicarbonate, and intravenous fluid repletion. He was also started on loperamide 4 mg four times daily, psyllium, and bismuth subsalicylate 262 mg three times daily, with improvement in his diarrhea, and was discharged for outpatient follow-up.

Subsequent hospitalization

The following day, the patient presented to the emergency department again for persistent hypotension, with systolic blood pressures in the 60s at home despite ongoing loperamide and psyllium. In the emergency department, he was afebrile with a heart rate of 57 beats per minute and blood pressure 94/47 mmHg. Labs showed improved electrolyte disturbance except for hypocalcemia and new hypoalbuminemia. Repeat infectious stool studies were negative (Table 1). Computed tomography (CT) enterography showed no evidence of enteritis. Olmesartan was held given the risk of olmesartan-associated enteropathy, although the patient had been taking this medication for over a year. Given the severity of his diarrhea, he was started on budesonide 9 mg daily for presumed refractory celiac disease versus olmesartan-associated enteropathy. Within two days of starting budesonide, his bowel movements became formed, and he no longer required loperamide.

Follow-up and final diagnosis

The patient's final diagnosis was celiac crisis, with a possible contribution from olmesartan-associated enteropathy. Budesonide was subsequently tapered and discontinued after resolution of his symptoms. Outpatient capsule endoscopy showed a normal small bowel without findings to suggest refractory celiac disease, and repeat celiac serologies showed improvement following treatment with a gluten-free diet and budesonide.

On subsequent push enteroscopy eight months after presentation, the visualized small bowel appeared normal without scalloping, fissuring, nodularity, or loss of folds typically associated with celiac disease. Biopsies from the jejunum and distal duodenum showed no histopathologic abnormality and no Marsh lesion, while the duodenal bulb showed only focal gastric foveolar metaplasia, again without Marsh lesion, consistent with histologic remission.

## Discussion

Celiac disease

Celiac disease affects approximately 1% of the general population, with a mean age of diagnosis of 38 years, although roughly 20% of patients are diagnosed after age 60 [5]. Diagnosis relies on a combination of compatible clinical features, positive celiac-specific serology, characteristic small-bowel histology, and clinical response to a gluten-free diet [5]. Tissue transglutaminase antibody testing has a sensitivity of 63-93% and specificity of 96-100%; a negative anti-tTG IgA carries a high negative predictive value when pretest probability is low to moderate, but symptomatic patients with high pretest suspicion should undergo upper endoscopy regardless of serologic results, with biopsies taken from the bulb and post-bulbar duodenum [6].

Classic celiac disease presents with a malabsorptive syndrome of diarrhea, steatorrhea, weight loss, or growth failure, but commonly patients present with non-classic disease, dominated by extra-intestinal features such as iron, B12, or folate deficiency, abnormal liver tests, or neurologic and psychiatric symptoms [5]. Histologically, celiac disease is characterized by increased intraepithelial lymphocytes, partial or total villous atrophy, crypt hyperplasia, and chronic inflammation of the lamina propria. Management centers on a strict, lifelong gluten-free diet [6].

When symptoms persist beyond 6-12 months of a gluten-free diet, this is termed nonresponsive celiac disease, most commonly due to inadvertent gluten exposure or an overlapping condition such as microscopic colitis [6]. Refractory celiac disease, defined as malabsorption and villous atrophy persisting beyond one year despite strict dietary adherence, is subdivided into type 1 (polyclonal, normal intraepithelial lymphocyte population) and type 2 (clonal, aberrant intraepithelial lymphocytes), the latter carrying an increased risk of enteropathy-associated T-cell lymphoma [7,8]. Open-capsule budesonide, which delivers drug throughout the small bowel rather than only the distal ileum and colon, has been shown in retrospective series to produce clinical response in 92% and histologic response in roughly 89% of patients with refractory celiac disease [9,10].

Celiac crisis

Celiac crisis is a rare, fulminant presentation of celiac disease characterized by severe diarrhea, dehydration, and metabolic disturbance, occurring in fewer than 1% of patients with celiac disease. It is thought to arise from severe mucosal inflammation, immune activation, and disrupted motility, and is often precipitated by an immune stimulus such as surgery, infection, or pregnancy in a patient with previously undiagnosed celiac disease. Diagnostic criteria require acute or rapidly progressive gastrointestinal symptoms attributable to celiac disease necessitating hospitalization or parenteral nutrition, plus at least two of the following: hemodynamic instability or orthostasis, neurologic dysfunction, renal dysfunction (creatinine > 2.0 mg/dL), metabolic acidosis (pH < 7.35), hypoproteinemia (albumin < 3.0 g/dL), electrolyte derangement, or weight loss exceeding 10 pounds [2].

Management of celiac crisis includes a gluten-free diet and aggressive nutritional support; a minority of patients require corticosteroid therapy. Response to steroids is typically rapid, with pediatric patients often showing improvement within 24 hours of initiation. Because the clinical course varies, the duration of corticosteroid therapy should be individualized and guided by ongoing clinical assessment rather than a fixed treatment length [2,11].

A 2010 case series of 12 patients with celiac crisis found a mean age of diagnosis of 58.9 years, with 11 of 12 patients diagnosed with celiac disease only at the time of the crisis [2]. All patients had elevated tissue transglutaminase IgA (except one patient with IgA deficiency) and Marsh 3 villous atrophy on biopsy. Roughly half of patients required corticosteroids, most commonly intravenous or oral prednisone tapered to budesonide. Most patients improved rapidly, within two weeks, after starting a strict gluten-free diet, although nutritional support or corticosteroid therapy was occasionally needed for up to 40 weeks in a minority of patients. A subsequent systematic review published in 2019 of 42 cases similarly found a median age of 50 years, with most patients diagnosed with celiac disease at the time of their crisis presentation [12]. Diarrhea was present in all cases, weight loss occurred in two-thirds, and there was a significant association with other autoimmune conditions. All cases had Marsh 3-4 lesions on biopsy. Outcomes were generally favorable with supportive care, gluten-free diet, and corticosteroids in a minority of cases.

Olmesartan-associated enteropathy

Olmesartan, an angiotensin II receptor blocker, is a rare but increasingly recognized cause of severe diarrhea with malabsorption and weight loss, with an estimated annual incidence of 1.3 cases per 1,000 individuals per year [13]. Symptom onset typically occurs 0.5-7 years (mean approximately 3.1 years) after starting the medication, and risk appears higher in older patients, those on therapy longer than one year, and those on higher cumulative doses [4]. The condition is associated with anemia, hypoalbuminemia, electrolyte imbalance, and vitamin deficiencies, and histology often shows duodenal villous atrophy, increased intraepithelial lymphocytes, or a thickened subepithelial collagen layer, sometimes with lymphocytic gastritis or colitis [4,14]. Treatment consists of discontinuing the offending medication. Clinical resolution typically occurs rapidly (within 48 hours to one week) with mucosal recovery expected over three to six months; immunosuppressive therapy is rarely required [14-16].

## Conclusions

Celiac crisis is a rare and often overlooked fulminant presentation of celiac disease, typically occurring in patients without a prior diagnosis, and is characterized by severe diarrhea, dehydration, and metabolic disturbances. It is commonly precipitated by an acute illness, surgery, or other immune stimulus, and management centers on a gluten-free diet, nutritional support, fluid resuscitation, and correction of metabolic derangements. Corticosteroids, including open-capsule budesonide, may produce a rapid response and are a reasonable option for celiac crisis. Olmesartan should be considered as a potential contributor to enteropathy in patients on angiotensin receptor blockers presenting with unexplained diarrhea and malabsorption. The prognosis after drug withdrawal is generally excellent.

In the case presented, celiac crisis was the leading diagnosis, supported by severe diarrhea, hypotension, AKI, metabolic acidosis, electrolyte abnormalities, weight loss, strongly positive serology, and compatible histology. However, concurrent olmesartan use and its withdrawal complicated the diagnostic picture. Nevertheless, the patient's symptoms persisted despite five days of olmesartan withdrawal and seven days of a gluten-free diet, but resolved rapidly after initiation of budesonide, suggesting that budesonide was an effective treatment in this setting.
